# Early Life Stress, Depression And Parkinson’s Disease: A New Approach

**DOI:** 10.1186/s13041-018-0356-9

**Published:** 2018-03-19

**Authors:** Ernest Dallé, Musa V. Mabandla

**Affiliations:** 1 School of Laboratory Medicine and Medical Sciences, College of Health Sciences, University of KwaZulu-Natal, Durban, 4000 South Africa; 2 School of Laboratory Medicine and Medical Sciences, College of Health Sciences, University of KwaZulu-Natal, Durban, 4000 South Africa

**Keywords:** Early stress, Depression, Dopamine, Fluvoxamine maleate, Parkinson’s disease

## Abstract

This review aims to shed light on the relationship that involves exposure to early life stress, depression and Parkinson’s disease (PD). A systematic literature search was conducted in Pubmed, MEDLINE, EBSCOHost and Google Scholar and relevant data were submitted to a meta-analysis. Early life stress may contribute to the development of depression and patients with depression are at risk of developing PD later in life. Depression is a common non-motor symptom preceding motor symptoms in PD. Stimulation of regions contiguous to the substantia nigra as well as dopamine (DA) agonists have been shown to be able to attenuate depression. Therefore, since PD causes depletion of dopaminergic neurons in the substantia nigra, depression, rather than being just a simple mood disorder, may be part of the pathophysiological process that leads to PD. It is plausible that the mesocortical and mesolimbic dopaminergic pathways that mediate mood, emotion, and/or cognitive function may also play a key role in depression associated with PD. Here, we propose that a medication designed to address a deficiency in serotonin is more likely to influence motor symptoms of PD associated with depression. This review highlights the effects of an antidepressant, Fluvoxamine maleate, in an animal model that combines depressive-like symptoms and Parkinsonism.

## Introduction

**Fig. 1:**
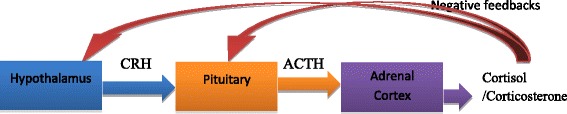
The HPA axis and the main hormones secreted by each gland in the axis. CRH: corticotropin releasing hormone. ACTH: adrenocorticotropic hormone. A stressor (e.g., a threat to the life of the organism) triggers the paraventricular nucleus of the hypothalamus to synthesize and secrete CRH, which binds to specific receptors in the anterior pituitary. This stimulates the synthesis and release of ACTH which is released into the circulatory system. ACTH triggers the synthesis and secretion of glucocorticoids (cortisol in humans/corticosterone in rodents) from the adrenal cortex

Stress is defined as a sudden inconsistent physical, physiological and social environmental change experienced by an organism [1–3]. Exposure to stress during early life can have short- or long-term effects on brain development, and these effects may include learning deficits and/or psychiatric disorders such as generalized anxiety and depression [4, 5]. The mechanism by which stress induces these psychological changes mainly involve the hypothalamic-pituitary-adrenal (HPA) axis [6, 7]. The HPA axis (Fig. 1) is a system that controls the organism’s response to stress and regulates certain circadian activities [8, 9]. In response to stress, the HPA axis induces the release of hormones (glucocorticoids and mineralocorticoids) by the hypothalamus, the anterior pituitary and the adrenal cortex [8, 10]. For instance, activation of the HPA axis stimulates the release of corticotropin releasing factor (CRF) from neurons in the paraventricular nucleus (PVN) of the hypothalamus which stimulates the release of adrenocorticotropic hormone (ACTH) from the anterior pituitary gland, which in turn, facilitates the release of cortisol/corticosterone release from the adrenal cortex [8, 10]. In addition, the HPA axis has negative feedback systems that prevent excessive hormonal secretion as well as prolonged stimulation of these systems [11]. Studies have shown that corticosterone plays a role in suppressing prostaglandin synthesis, modulates the immune response and exerts negative feedbacks inhibition on hormone release in the hypothalamus and the anterior pituitary gland [8, 10, 12]. Early life stressors such as prenatal maternal stress, early postnatal maternal separation, early postnatal stress or early social isolation have been implicated in the development of psychiatric disorders as they can affect brain development and hence behavior over time [13, 14]. Studies have demonstrated that exposure to early maternal separation increased plasma ACTH and corticosterone levels in the adult offspring suggesting hyperactivation of the HPA axis [15–19]. High corticosterone levels may, therefore, give rise to a blunted stress response due to desensitization of glucocorticoid (or mineralocorticoid) receptors at different levels of the HPA axis preventing efficient negative feedback [18].

## Maternal separation

Early social interactions including attachment between the mother and the offspring are known to be critical for social behavior and normal physiological development [20, 21]. The rodent maternal separation model is widely used to study the effects of early environmental exposure to stress on physiological and behavioral functions later in life [18, 22–27]. This stress model originates from a study that showed that daily brief handling (10 minutes) of pups can result in fearfulness in adulthood [28]. Other studies showed that 3 minutes of daily maternal separation of the pups from the dam reduced the physiological response to stress [29, 30]. Different maternal separation stress protocols (with varied duration and number of days of separation) have been used in laboratory animal studies to investigate the short- or long-term behavioral effect of stress in early life. The duration of separation may be seen as short when not exceeding 15 minutes or long when it lasts for 3 hours or more [18, 27, 31]. Short-term maternal separation mainly evaluates the protective response to the stressor while long-term separation may evaluate the environmental factors affecting normal neurobiological development [32]. The duration and number of separations (single or repeated) is critical as studies have shown that pups that are separated from their dam during the stress hyporesponsive period (post-natal day 2 to post-natal day 14) exhibit increased anxiety and/or depressive-like behavior later in life [18, 33–36]. The stress hyporesponsive period is crucial for the protection of the developing brain from high glucocorticoid levels that have been associated with abnormal neural and behavioral functions since it can result in a neuropathological condition such as depression [31, 34].

## Depression and Anxiety

Clinical depression also called major depression or major depressive disorder (MDD) is a mental state characterized by loss of pleasure or interest in almost all activities [37, 38]. Based on symptomatic criteria, depression cannot be viewed as a single disorder but as a heterogeneous syndrome comprised of several symptoms of distinct causes and pathophysiology [37]. Anxiety disorder is also a psychological disorder with similar symptoms found in patients with depression although the difference is that anxiety may precede depression in most patients [39]. The overlap of symptoms associated with depression and anxiety sometimes makes the diagnosis, research, and treatment difficult [40]. Depression and anxiety are often described as stress-related disorders as they occur in the context of some form of chronic stress or emotional trauma experienced in life [5, 41, 42]. They may, therefore, be felt as major pathological changes that progressively affect the brain’s structure via abnormal hormonal secretion, and eventually affect the mental health [5]. In addition, chronic stress experience usually causes post-traumatic stress disorder (PTSD) which may be totally different from depression in terms of the symptoms, treatment and even the longitudinal course of the disease [37]. As the stress mechanism leading to these psychiatric disorders are still not well understood, we reviewed the literature to investigate the neurobiology of stress leading to depression in a new perspective.

## Pathophysiology of stress leading to depression

Although there are a number of hypotheses about stress leading to depression, most include a change in the concentration of neurotransmitters such as dopamine, epinephrine, glutamate, γ-aminobutyric acid (GABA), noradrenaline and serotonin [43, 44]. Neurotransmitters are biochemical substances that relay signals between nerve cells within the brain and the body [45]. Most neurotransmitters can be both excitatory and inhibitory depending on the receptors they activate [46, 47]. Dopamine is a catecholamine modulatory neurotransmitter which can be both inhibitory and excitatory [48]. Studies have shown that low levels of dopamine are associated with Parkinson’s disease (PD) [49, 50]. Noradrenaline/norepinephrine is also a catecholaminergic neurotransmitter which is involved in mood, motivation, emotion and cognitive functions [51]. Studies have shown that norepinephrine deficiency is associated with depression [52]. Serotonin is a monoamine neurotransmitter that is needed for maintenance of a stable mood [43, 48]. Studies have linked serotonin depletion to depression [43, 48, 53]. Therefore, altered neurotransmitter release may correlate in some way with the development of a number of psychiatric disorders including depression. Since stress may disturb brain circuits or neural pathways that convey the signals of these neurotransmitters, it follows that HPA axis dysfunction may cause hormonal imbalances, behavioral deficits and/or mood disorders [54, 55]. This may suggest a key role of the HPA axis in the development of neuropsychiatric disorder including depression [54–56]. In the present review, we focused on 3 hypotheses that propose that stress disturbs neuronal processes and leads to depression. These are:

### Noradrenaline hypothesis of depression

Noradrenaline is known to play a role in the regulation of emotions [44]. The deficiency of noradrenaline/norepinephrine mainly produced in the locus coeruleus and affecting certain brain areas such as the prefrontal cortex, the hippocampus or the hypothalamus has been associated with depression [57–59]. Studies have shown that exposure to chronic stress including early maternal separation decreases noradrenaline levels within the brain leading to depression [60, 61]. This explains why selective norepinephrine re-uptake inhibitors (SNRIs), a new class of antidepressants that work by increasing norepinephrine levels in the brain, have been used to treat depression [58].

### Serotonin (5-HT) hypothesis of depression

Serotonin (5-HT) is mainly produced in the dorsal raphe nucleus [62]. Serotonin transporters take up released serotonin from the synaptic cleft into serotonergic neurons in a manner that helps to modulate various functions in the brain including mood and emotion [58]. The striatum, the amygdala, and the prefrontal cortex are regions of the brain that are innervated by serotonergic neurons [58]. These brain regions including the dorsal raphe nucleus which is part of the brain’s serotonergic system, are activated during early maternal stress [59]. Abnormal 5-HT levels in these brain areas have been associated with depression [55, 59, 63]. Pre-clinical and clinical studies have demonstrated that early life stress affects 5-HT levels in the brain and this may lead to depression [5, 55, 59, 61, 64–66]. Selective serotonin re-uptake inhibitors (SSRIs) are a class of antidepressant drugs commonly used to treat depression [55, 67, 68]. SSRIs work by blocking 5-HT re-uptake thus increasing the availability of 5-HT in the synaptic cleft as well as its chance to bind to receptors in the post-synaptic membrane [69]. Therefore, by restoring the levels of monoamines and their transporters in the brain, SSRIs drugs are appropriate treatments to address early life stress dysfunction that predisposes to depression later in life.

### Dopamine hypothesis of depression

Dopamine is produced in the substantia nigra pars compacta in the midbrain. Dopaminergic projections in both the mesocortical and the mesolimbic systems are known to be disturbed by stress [59, 70]. Dopaminergic pathways are part of the reward system and the effects of chronic stress on reward perception that lead to depression can occur because of the interaction between the dopaminergic system and the HPA axis and between the dopaminergic system and the serotonergic system [71, 72]. Studies have demonstrated that early psychological stress that activates the HPA axis, exacerbates DA depletion and is associated with a decrease in DA synthesis in the brain [5, 59, 65]. Auffret *et al*. [73] and Leentjens, [74] have shown that symptoms of depression can be improved by administration of DA agonists highlighting the possibility of antidepressant drugs to have an affinity to DA receptors. Since DA depletion may accompany depression, some antidepressant drugs (SSRIs or SNRIs) may act on both dopaminergic and serotonergic systems to exert their antidepressant effect [75–78]. Therefore, DA deficiency resulting from early life stress may in some instances predispose an individual to depression and eventually to neurodegenerative diseases such as PD.

## Parkinson’s disease (PD)

Parkinson’s disease (PD) is a neurodegenerative disorder characterized by selective degeneration of dopaminergic neurons in the nigrostriatal pathway resulting in DA deficiency in the substantia nigra pars compacta [79–81]. In addition to being the most common movement disorder, PD is the second most prevalent neurodegenerative disorder in the western world after Alzheimer’s disease [82–84]. PD has a prevalence of 1 in 100 people over the age of 50 [84, 85]. The diagnosis is usually made in the sixth or seventh decade of life although rare cases are found in people in their forties [76]. The major motor symptoms associated with the pathogenesis of PD are: resting tremor (shaking of a body part when at rest), rigidity (resistance to movement when trying to move), akinesia (absence of normal unconscious movements), bradykinesia (slowness of movement), hyperkinesia (reduction in movement amplitude), and postural instability (impaired balance of the body) [86–89]. These major motor symptoms are frequently accompanied by non-motor symptoms including anxiety, depression and/or impairment in cognitive functions where patients are more likely to develop frank dementia early or late after diagnosis [90–92].

PD is progressive and primarily affects the basal ganglia which are involved in voluntary motor control [80, 93–95]. The basal ganglia are a group of nuclei made up of the caudate nucleus, the putamen, the substantia nigra (pars compacta and pars reticulate), the subthalamic nucleus and the globus pallidus forming a network interconnecting them to one another so as to process chemical signals that initiate or terminate movement [80]. Studies have also shown that the progressive degeneration of dopaminergic neurons causes dysregulation of the motor circuits that project throughout the basal ganglia, resulting in clinical manifestations of PD [79, 82, 96–99]. In the basal ganglia, the substantia nigra pars compacta contains the dopaminergic neurons which degenerate in the course of PD [100]. Although the cause of the degeneration is not well established, it has been suggested that dopaminergic neurons have a physiological phenotype that may contribute to their vulnerability and eventually to their death [101]. For instance, it has been shown that monoamine oxidase (MAO) dysfunction causes free radical production which in the case of PD may result in DA being subject to auto-oxidation thus favoring cell death by apoptosis [80, 102].

The motor symptoms of PD are secondary to the neuronal degeneration that occurs in the central nervous system several years prior to the onset of the clinical symptoms [95]. Numerous studies have shown that PD is characterized by a long preclinical phase which frequently includes depression symptoms [85, 95, 103, 104]. However, compensatory mechanisms in the nigrostriatal pathway or outside the basal ganglia may slow down neuronal loss until exogenous events exacerbate the loss of dopaminergic neurons and symptoms appear [102, 103, 105]. It is now clear that this silent phase of the disease may hide a cascade of events that are important in both the aetiology and the diagnosis of the disease [106, 107]. Therefore, a critical look at this asymptomatic phase may not only help to predict the disease, but will also help to monitor people at risk of developing the disease. While PD is still mainly an idiopathic disease, studies have shown that it can also have a genetic or an environmental origin, and both origins can be associated with early life stress [5, 59, 87, 108–110].

## Genetic causes of Parkinson’s disease (PD)

Although the genetic causes of PD can only account for a few percentage of patients with genes and genetic loci dysfunctions, early stress exposures usually predispose to depression, prompt or worsen motor symptoms in PD [87, 111]. The genetic origin of PD mostly includes mutations in α-synuclein, parkin phosphatase and tensin homologue (PTEN)-induced putative kinase 1 (PINK), leucine-rich repeat kinase 2 (LRRK-2) and DJ-1 (protein deglycase) genes [112–115]. Mutations of these genes can result in abnormal protein accumulation, α-synuclein aggregates, protein phosphorylation, mitochondrial dysfunction and oxidative stress (from exogenous stressors and/or endogenous neurotoxins) which is the most common pathway to cell death in PD pathogenesis [82, 96]. As exposure to stress early in life is known to alter both the behavior and physiology in certain brain areas, it is accepted that genetic changes associated with PD are in part the result of alterations in gene expression in these brain areas also affecting the stress response system [5, 59, 109].

α-Synuclein (encoded by the autosomal dominant gene α-synuclein) is a core component of Lewy bodies or Lewy neurites in all patients with PD [116]. Postmortem analysis of the brains of individuals that had PD has shown neuronal inclusion of Lewy bodies or Lewy neurites [117, 118]. It has been shown that Lewy bodies and Lewy neurites accumulate in the substantia nigra pars compacta during endogenous activity prior to the manifestation of the disease [118]. Lewy bodies may then cause toxicity by the activation of cell death pathways [114]. Therefore, it is more likely that, stress will result in abnormal protein processes enhanced by changes in neurochemical systems, exogenous or endogenous factors leading to cell death within the brain thus play a key role in the aetiology of PD [109].

Studies have also shown that the parkin and PINK genes are implicated in the development of PD [114, 119–121]. Mutations in the parkin gene are common in familial PD patients with early disease onset (before the age of 45) [116, 122]. The parkin protein is involved in regulation of gene transcription and translation, DNA repair as well as in the removal of damaging protein aggregates resulting from oxidative stress [112, 114, 119]. Mutations in this gene may, therefore, cause protein accumulation and eventually promote the development of PD. The PINK protein is implicated in the translocation of parkin to the mitochondria [112, 114]. Studies have shown that PINK is involved in the mitophagy of dysfunctional mitochondria [112, 114]. Mutations in this gene disrupt the integrity of the mitochondria and may result in oxidative stress and eventually to neuronal damage [112, 114]. Altogether, this implies that PD genetic mutations increase cell susceptibility to stress and favor cascade of events that are known to interfere with the stress response system.

Mutations in LRRK-2 are the most common cause of autosomal dominant PD [116, 123]. LRRK-2 mutation promotes an increase in its kinase activity which may result in dopaminergic neuronal loss and gliosis in the substantia nigra [114, 116]. The evidence linking *LRRK-2* to pathogenic mechanism of PD such as α-synuclein, tau, inflammatory response, oxidative stress, mitochondrial dysfunction, synaptic dysfunction as well as autophagy-lysosomal system is known [124]. LRRK-2 mutations can unable the organism to efficiently respond to these events and have been connected to the early onset of PD where patients exhibit pre-motor symptom such as depression [125–127].

DJ-1 functions as a redox sensor and protects against oxidative damage [59, 112, 114, 119]. DJ-1 also acts as a direct free radical scavenger by regulating the ASK1/Trx1 (apoptosis signaling-regulating kinase 1/thioredoxin 1) complex which is known to be involved in mediating oxidative damage [59, 112, 114, 119]. It has been shown that exposure to stress plays a role in the modification of genetic composition by altering epigenetic mechanisms thus leading to altered gene expression [128]. Therefore, in PD, it is accepted that under stress circumstances, DJ-1 gene mutations may result in mitochondrial dysfunction and hence increased susceptibility to oxidative stress which exacerbates neuronal cell death [59, 128].

## Environmental toxins and Parkinson’s disease (PD)

Neuronal cell death in PD may also be triggered by exposure to toxic substances or environmental factors which precipitate the symptoms of the disease as they render the brain vulnerable to subsequent physiological chronic stress [87, 129–131]. The environmental cause of PD mainly refers to exposure to dopaminergic toxins 6-hydroxydopamine (6-OHDA), 1-methyl-4-phenyl-1,2,3,6-tetrahydropyridine (MPTP), paraquat and rotenone as these toxins are known to induce formation of reactive oxygen species (ROS) and oxidative stress which may result in neuronal cell death [87, 132, 133].

DA is one of the common neurotransmitters present in most parts of the central nervous system [71]. The mesocortical, mesolimbic, nigrostriatal and tubero-infundibular pathways are the four main pathways that play a key role in dopaminergic signaling [71]. DA cannot cross the blood brain barrier, therefore, it is synthesized from tyrosine which is carried into the brain via amino acid transporters [71, 80]. At the dopaminergic neuron level, tyrosine is then converted into dihydroxyphenylalanine (L-DOPA) by tyrosine hydroxylase (TH) then finally into DA by aromatic L-amino acid decarboxylase (AADC) [71]. DA is then stored in the vesicle until an action potential allows the vesicle to be discharged into the synapse [71]. Monoamine oxidase (MAO) is the enzyme that is responsible for breaking down excess DA and is known to similarly act on 6-OHDA inducing oxidative stress resulting in apoptosis [102].

In animal studies, to create a parkinsonian rat model, an intracerebral injection of 6-OHDA into the medial forebrain bundle (MFB) has been widely used to study the destruction of dopaminergic neurons in the nigrostriatal pathway [27, 35, 134]. This model has also been used to evaluate the effects of pharmacological agents in ameliorating motor deficits associated with PD [88]. Some studies have even shown that in a 6-OHDA parkinsonian rat model, prenatal or early postnatal stress exacerbated the neurotoxic effect of 6-OHDA [19, 27, 35, 36, 135, 136]. The neurotoxin 6-OHDA may cause neuronal cell death by two main pathways. Firstly, it can enter the mitochondria, and inhibit mitochondrial complexes I and IV of the mitochondrial respiratory enzymes resulting in impairment of neuronal function [80, 105]. Secondly, 6-OHDA can accumulate within the cytosol causing auto-oxidation which will result in the formation of reactive oxygen species and oxidative stress and thereafter will cause neuronal death by apoptosis [80, 102].

Other toxins used to mimic PD include MPTP. MPTP has been shown to produce symptoms nearly identical to PD in humans and non-human primates [87, 137]. MPTP was formed from an analog of the narcotic meperidine during an unconventional preparation of 1-methyl-4-phenyl-4-propionoxypiperidine (MPPP) [137]. Under stress conditions, clinical studies have shown that MPTP can cross the blood brain barrier and accumulate in the mitochondria, the cytosol and the synaptic cleft [87, 132]. MPTP is then oxidized to 1-methyl-4-phenyl-2.3-dihydropyridinium (MPDP+) by MAO-B in glia and serotonergic neurons and thereafter to MPP+ [87]. Inside the neuron, MPP+ can firstly bind to the vesicular monoamine transporter-2 (VMAT-2) which translocates MPP+ into the synaptosomal vesicles facilitating interactions in the synaptic cleft [87, 132, 138]. Secondly, MPP+ can accumulate within the mitochondria causing alterations of complex I of the mitochondrial respiratory chain by decoupling oxidative phosphorylation and the electron transport chain [87, 139]. Thirdly, MPP+ can also remain in the cytosol to interact with cytosolic enzymes thereby limit access to the mitochondria as a possible neuroprotective strategy [87, 140].

Paraquat (N, N’-dimethyl-4-4’-bipiridinium) is an herbicide present in the environment which can also cause symptoms of PD in humans and non-human primates [87]. Paraquat has a structural similarity to MPP+ although it cannot cross the blood brain barrier easily [141]. Repeated exposure to paraquat sometimes results in the formation of superoxide radicals that may result in dopaminergic neuron degeneration in the substantia nigra pars compacta together with α-synuclein accumulation in some brain areas such as the frontal cortex [87, 142, 143].

Rotenone is a cytotoxic compound extracted from tropical plants, which is widely used as an insecticide and fish poison [87]. Rotenone, a lipophilic pesticide, binds at the same site as MPP+ and inhibits complex I at the mitochondrial level [87]. This results in selective degeneration of nigrostriatal dopaminergic neurons with α-synuclein-positive Lewy body inclusions [87, 144]. Altogether, it has become apparent that both the genetic and the environmental factors associated with PD can be influenced by stress and lead to complications. However, as all the above-mentioned factors produce oxidative stress, increased levels of oxidative stress in the brain is critical for the initiation and development of neurodegeneration and can contribute importantly to reducing the pathophysiological dysfunctions in PD.

## Oxidative stress, neuroinflammation and Parkinson’s disease (PD)

Oxidative stress is the result of an imbalance between the production of reactive oxygen species (free radicals) and the body capacity to counteract their harmful effects through neutralization by antioxidant defenses [145]. Brain neurons are constantly exposed to reactive oxygen species and reactive nitrogen species as a result of endogenous or exogenous exposure to oxidative stress [146]. Chronic psychological stress increases neuroinflammation which may facilitate nigral cell death in PD [19, 36]. For instance, under stress conditions, there is evidence that dysfunction of inflammatory markers such as tumor necrosis factor (TNF)-α, interleukin (IL)-1β, IL-6, IL-10, transforming growth factor (TGF)-β in microglia (the major resident immune cells in the brain) of patients with depression participates in worsening PD symptoms [53, 59, 146].

In PD, plus oxidative damage to lipids, proteins, and DNA, oxidative damage such as 4-hydroxynonenal (HNE), can react with proteins to impair cell viability [146, 147]. Oxidative stress can then contributes to the cascade leading to DA cell degeneration through mitochondrial dysfunction, excitotoxicity, nitric oxide toxicity and inflammation [147]. For example, due to its high lipid composition, the brain is highly susceptible to lipid peroxidation [146]. There is evidence of lipid peroxidation in the substantia nigra induced by high levels of trace elements such as ferrous iron which can exacerbate cell damage in PD [146, 148]. Studies have also shown that other trace elements such as manganese, selenium, copper, aluminum or zinc also play a role in neurodegeneration [149, 150]. Their abnormal metabolism sometimes results in pathological conditions including depression and PD [149, 150]. Moreover, studies have shown that oxidative stress caused by abnormal levels of these trace elements may increase the risk of exacerbation and/or neuronal death seen in most neurodegenerative disorders [146, 148, 151]. In a new perspective, since oxidative stress plays a key role in dopaminergic neurotoxicity, lipid peroxidation may, therefore, be an intermediate step in the neurodegeneration observed in PD associated with depression.

PD research is often directed towards the prevention of DA neuron degeneration [19, 87, 136, 152]. However, all current treatments only address the symptomatic effects of the disease, none of which neither halt nor retard DA neuron degeneration [87]. About 95% of PD cases are sporadic hence caused by environmental factors versus 5% that are inherited (familial) [87, 108]. The point of view in favor of exposure to stressful events early in life predisposing an individual to develop neurodegenerative disorders later in life seems to emphasize that PD is much more than just a DA-dependent motor deficit.

## Role of serotonin (5-HT) in Parkinson’s disease (PD)

Studies have shown that the 5-HT transmission system also undergoes degeneration in PD [153, 154]. The neuronal degeneration in the midbrain raphe nuclei (where 5-HT is mainly produced) is known to lead to reductions in 5-HT and 5-HT transporter levels in brain areas such as the striatum and prefrontal cortex [153, 155]. However, 5-HT neurons have the ability to store and release DA synthesized from systematically administered DA medication such as levodopa [156, 157]. For instance, in a 6-OHDA lesioned rat model of PD with severe nigrostriatal dopaminergic neuron degeneration, it has been shown that striatal reuptake of levodopa-derived DA can occur through 5-HT transporters [78, 158]. Further, it has been shown that monoamine transporter inhibitors such as selective serotonin reuptake inhibitors (SSRIs) can modify striatal dopamine reuptake and metabolism so as to improve motor symptoms of PD [159]. A new treatment approach for PD may therefore consist of blocking 5-HT transporters to enhance and/or prolong the antiparkinsonian effects of drugs that have the potential to increase extracellular DA in the striatum including SSRIs.

It has been established that acute and chronic stress may cause a significant elevation in serotonergic and/or noradrenergic activity in areas of the brain such as the prefrontal cortex, striatum, and hippocampus [59, 76, 160–163]. The altered serotonergic transmission that has been implicated in a number of non-motor and motor symptoms of PD may also be the result of weak neuromodulation by the DA neurons [156]. Zou *et al*. have shown that emotional stress may transiently increase motor symptoms and reveal damages to the nigrostriatal pathway that have been masked during the preclinical stage of PD [76]. In early onset PD, since stress increases the extracellular availability of DA and 5-HT (all these have the capability to harm neurons separately or synergistically), stress may cause or exacerbate neuronal damage [5]. 5-HT loss and non-motor symptoms (depression, anxiety, sleep disorder, mood and emotional disorders) associated with PD may then result from the synergistic action of the serotonergic and dopaminergic systems [163–165]. The possibility that depression is a harbinger of PD and that treating depression may help in the management of Parkinsonism is therefore of great importance.

## Non-motor symptoms associated with Parkinson’s disease (PD)

Studies have shown that the pathophysiology of PD goes beyond the basal ganglia and results in non-motor dysfunction coexisting with motor symptoms [166]. Evidence has emerged suggesting that the burden of non-motor symptoms may negatively impact the quality of life of patients with PD [167–170]. There are a number of non-motor symptoms associated with PD; however, few studies have investigated their role in the disease process. These non-motor symptoms have been under-reported thus frequently not treated by clinicians [170]. Chaudhuri and Schapira showed that up to 62% of non-motor symptoms in PD may remain undeclared to health-care professionals because patients are either embarrassed or unaware that the symptoms may be linked to PD [171, 172]. Non-motor symptoms commonly associated with PD include a range of symptoms from neuropsychiatric to autonomic dysfunctions where the most cited are depression, anxiety, apathy, pain, bowel incontinence, sleep disorder and most recently erectile dysfunction [10, 59, 169, 170, 172–175].

This review focuses on depression and/or anxiety as they are early non-motor symptoms of PD [59, 171, 174, 176]. These two non-motor symptoms associated with PD can be managed by various drugs including antidepressants [171]. Awareness that depression and/or anxiety may be related to PD is vital as research for treatment and causation may be the cornerstone for delivering a comprehensive modern treatment tool for these two disorders [177].

## Treatment of depression/anxiety associated with Parkinson’s disease (PD)

Antidepressants are a popular treatment for moderate to severe forms of depression in PD [178–182]. Several classes of antidepressants are available and they work in a slightly different way with different side-effects. Neurotransmitters (DA, 5-HT, and norepinephrine) are associated with the pathogenesis of depression in PD [43, 183, 184]. Antidepressants relieve the symptoms of depression by targeting these neurotransmitters [58, 74, 78, 157, 159, 185]. Tricyclic, monoamine oxidase inhibitors (MAOIs) and newer selective antidepressants including serotonin and noradrenaline reuptake inhibitors are classes of antidepressants known to be effective in treating depression [174, 186].

Tricyclic drugs are the older version of antidepressants [187–189]. This class of antidepressant includes drugs such as desipramine, doxepin, imipramine, trimipramine [190–192]. Although tricyclic antidepressant drugs are effective in treating depression, they have several side effects such as dry mouth, constipation, difficulty urinating, sedation, weight gain or sexual problems [193, 194]. Also, tricyclic drugs can be fatal in overdosing [187, 194].

MAOIs are also a class of antidepressant drugs prescribed to elevate norepinephrine, DA and 5-HT concentration in patients with depression [195–197]. MAOIs include selegiline, phenelzine or tranylcypromine [198]. They work by inhibiting MAO which breaks down monoamine neurotransmitters [199]. MAOIs are usually prescribed as last resort to patients due to their dangerous side-effects [200]. For example, combined with certain food or other medications, MAOI drugs can be fatal for the patient as they elevate blood pressure [200].

Selective serotonin reuptake inhibitors (SSRIs) and other noradrenaline reuptake inhibitors (SNRIs) are amongst the newer types of antidepressant drug used to treat depression [201]. These two classes of antidepressants have been reported to be better tolerated as they have minor side-effects in comparison to the older type of antidepressant classes such as tricyclics and MAOIs [174]. SSRIs are considered to be the first line treatment for depression and anxiety disorders [68]. Studies have shown that most patients with depression have a trait abnormality of 5-HT function and that treatment with SSRIs may compensate for such deficit [55, 68, 197].

SSRIs work by blocking the reuptake of the neurotransmitter 5-HT into the presynaptic neuron thus increasing the level of 5-HT in the brain [68]. Basically, their mechanism of action consists of increasing the level of 5-HT released from the presynaptic neuron into the synaptic cleft to act on the post-synaptic neuron [68]. When 5-HT is released from the presynaptic neuron into the synaptic cleft, it binds to receptors on the post-synaptic neuron and it undergoes reuptake into the presynaptic neuron by transporters [69]. Studies on depression, have demonstrated that the already reduced 5-HT levels present in the synaptic cleft, is quickly taken up by the presynaptic neuron [68, 202]. This suggests that these 5-HT transporters may be hindering binding of 5-HT to the post synaptic neuron [68, 197, 202]. Therefore, by antagonizing or blocking the 5-HT transporters using SSRIs, the serotonergic activity is altered thereby restoring chemical imbalance and relieving the symptoms of depression [68].

## The case of Fluvoxamine maleate

Commonly advertised under its commercial name of Luvox, Fluvoxamine maleate is a potent SSRI with little or no noradrenergic or anticholinergic activity [203]. Originally developed as an antidepressant, Fluvoxamine maleate is widely used as first-line treatment for major depressive disorders and/or anxiety disorders [69, 204–208]. Fluvoxamine maleate functions as a SSRI drug and a sigma 1 receptor (σ_1_R**)** agonist. The sigma 1 receptor is a chaperone protein in the endoplasmic reticulum that modulates calcium signaling through inositol 1,4,5-triphosphate (IP3) receptors [209]. Fluvoxamine maleate acts on the serotonergic reuptake pump by blocking reuptake of 5-HT and thus increasing the availability of 5-HT in the synaptic cleft which increases the probability of 5-HT binding to its post-synaptic membrane receptors (5-HT 1A, 5-HT 2, 5-HT 3, 5-HT 4) [69, 208]. This drug is well tolerated by the body, does not lead to adverse effects in overdose conditions and has no significant effect on body weight or on the cardiovascular system in patients as opposed to other antidepressant drugs [69, 207, 208, 210–213]. However, in patients with PD, it is possible that minor and infrequent side effects of Fluvoxamine maleate such as constipation, dizziness, diarrhea, excessive sweating, dry mouth, excess urination, muscle pain, worsen motor symptoms and extrapyramidal side effects may appear [69, 207]. Studies have also mentioned the serotonin syndrome (resulting from a multitude of serotonergic drug combinations that work by different mechanisms) which may worsen PD symptomatology. Common symptoms of the serotonin syndrome include nausea, headaches, shivering, confusion, high fever, seizures, tremor, twitching muscles [69, 207].

The role of serotonergic drugs in PD associated with depression has been receiving considerable attention amongst the research community [171, 214–217]. As a link between DA and the development of depression in patients with PD has been suggested, the pathophysiological features of both PD and depression have in common DA pathway dysfunction and depletion and/or 5-HT deficit [10, 36]. It has been suggested that an increase in serotonergic tone may indirectly influence DA function and may contribute to increased motor activity which is partially blocked by DA antagonists [77, 78, 218]. Studies have shown that depression may be associated with an abnormal level of DA [59, 70, 219]. As studies have also shown that brain regions affected by abnormal DA processing may also be affected when 5-HT is abnormally processed, we hypothesize that Fluvoxamine maleate treatment may play a role in improving the chemical imbalance caused by low levels of DA in the brain [219–223].

## Depression and Parkinsonism in an animal model of neurodegeneration

Early post-natal maternal separation is widely used to create an animal model that exhibits some depressive/anxiety-like behaviors [224, 225]. This established model of depression is useful to study 6-OHDA lesion of the medial forebrain bundle to lesion nigrostriatal DA neurons. We recently investigated the antiparkinsonian effects of Fluvoxamine maleate in a parkinsonian rat model of neurodegeneration associated with anxiety/depressive-like behaviors [36, 136]. Although these studies were a small exploratory open-label trial, they anticipated outcomes on a larger double-blind placebo-controlled study that include non-depressive animals with Parkinsonism. Fluvoxamine maleate treatment has shown potential in decreasing dopaminergic neuronal loss as well as potential to regulate neuronal pro- and anti-inflammation markers in the striatum [36, 136]. Therefore, a combined animal model of chronic stress-induced depression with a 6-OHDA lesioned parkinsonian animal model is an appropriate model to investigate the relationship between depression and PD. This association suggests that the stressor needs to be applied prior to the injection of the neurotoxin 6-OHDA to combine depressive-like behaviors with a potential risk of developing motor-symptoms that characterize Parkinsonism. This combination showed the double advantage of investigating a non-motor symptom (depression) as part of an early onset of PD together with the neuroprotective effects of a treatment on the development of the disease.

## Conclusion

Stress may trigger the symptoms of neurodegenerative diseases and expose dysfunctions that may have started many years ago [5, 59, 76]. Delaying the development of neurodegenerative diseases by addressing their long preclinical phase with an antidepressant such as Fluvoxamine maleate can be a new approach and/or an alternative way of increasing the life expectancy of those at risk of developing PD. Depression symptomatology is linked to dysfunction of the HPA axis which plays a key role in the development of neurodegenerative disease such as PD [56]. However, although psychosocial factors and disability are not always the predominant determinants of PD, depressive symptoms may be a relevant psychological reaction to the disease development and progressive disability of patients with PD. Therefore, irrespective of whether depression is an early symptom of PD or depression is a risk factor for PD, an individual who is depressed engages in behavior that is more likely to result in PD.
